# Meeting the eye-care needs of Australia’s Indigenous people

**DOI:** 10.2471/BLT.18.021018

**Published:** 2018-10-01

## Abstract

Australia is addressing the huge unmet need for eye care for its Indigenous population. Jack Latimore reports.

When Levi Lovett was 14 years old, he often struggled to catch the cricket ball during games.

At school, things weren’t much better. Growing up as a young Gunditjmara man in the south-eastern Australian state of Victoria, he spent most of his school years copying other kids’ work.

“I learnt more from listening and trying to understand the words, instead of reading the writing on the chalkboard,” he says. “If there wasn’t anyone close enough for me to look at their notes, I wouldn’t write it down.”

In his mid-20s, Lovett was finally able to correct his vision, when he underwent surgery on both his eyes. 

For the last four years Lovett coordinated eye health programmes at the Victorian Aboriginal Community Controlled Health Organisation. 

Lovett, who is now the Aboriginal Inclusion Coordinator with the state government, vividly remembers his father’s struggle with vision loss until he too had cataract surgery in his early 30s. He believes the cause was congenital hereditary cataracts, as the condition affected his father, sister and niece at an early age.

“After my father had his surgery for cataracts, he got a salad roll for lunch and he just sat there and stared at it for half an hour. Everyone was asking, ‘what’s going on?’, and he said, ‘I didn’t realise how bright and colourful salad sandwiches are’,” Lovett says.

When Lovett had surgery, the biggest shock was in the waiting room, when he saw how the other patients were so much older than him. “So what are you in for?” people asked. “I said, ‘cataracts’ and they replied ‘isn’t that an old person’s condition?’”

Australia’s Aboriginal and Torres Strait Islander people – who constitute the country’s Indigenous population – have significantly poorer health outcomes than the rest of the population.

Since 2006, Australia’s top health bodies, nongovernmental organizations (NGOs) and human rights organizations, have been working together to close the health and life expectancy gap within a generation by targeting preventable conditions. 

In 2008, the National Indigenous Eye Health Survey showed that the prevalence of blindness among Indigenous adults at 40 years of age was six times higher than that of other Australians of the same age and the prevalence of vision loss was three times greater. Vision loss, also known as visual impairment, is when a person loses the ability to see to the extent that they cannot be helped with glasses. 

The survey also found that 94% of the vision loss could have been prevented or treated with routine surgery, basic washing facilities, regular eye checks or glasses.

The main causes of vision loss were un-operated cataracts, diabetic eye disease, refractive error and trachoma.

“I learnt more from listening and trying to understand instead of reading the writing on the chalkboard.”

Levi Lovett

The survey also showed that 21% of Indigenous children had trachoma, an infectious disease that is not prevalent in any other developed country in the world.

Faye Clarke, an Aboriginal woman from the Gundatjmara, Wotjobaluk and Ngarrindjeri peoples, and a care coordinator at the Ballarat and District Aboriginal Co-operative in regional Victoria, says the impact of vision loss on the community is significant and wide reaching.

“It affects your income and your sense of identity, who you are and how you cope in the world,” Clarke says. “Particularly for Aboriginal people”, she adds, who don’t want to be seen as reliant on welfare as a result of vision loss.

Recognizing the unmet need for eye care for the Indigenous population and that previous attempts to address it have failed, a *Roadmap to close the gap for vision* *by 2020* was developed.

The roadmap, led by ophthalmologist Professor Hugh Taylor, head of the University of Melbourne’s Indigenous Eye Health Unit, contains 42 policy recommendations.

The roadmap’s aim is to eliminate preventable vision loss and bridge the vision loss gap, which accounted for 11% of the difference in health outcomes when the roadmap was launched in 2012.

“We’ve got universal health coverage in our country,” he says, adding that the health outcomes of the Indigenous people are not what you would expect in a high-income country. 

Taylor says the roadmap – which views the patient journey as a “leaky pipe,” where people fall through the system – takes a comprehensive health systems approach to addressing issues particularly around the lack of access and utilization of eye-care services.

The roapmap’s recommendations focus on integrating eye-care service provision into primary health care; enhancing access to eye health services; improving co-ordination of eye-care services and referral pathways; and boosting the eye health workforce, among others.

Taylor says 16 of 42 of the roadmap’s recommendations had been fully implemented and that progress to implement the remaining ones was being made with two-thirds of the intermediate steps completed. The goal is to implement all 42 recommendations by 2020. 

In 2016, the National Eye Health Survey found that the gap between Indigenous and other Australians’ rates of blindness had halved since 2008 and was three – rather than six – times greater.

For Taylor, the improvement was due to increased availability and use of services; increased provision of cataract surgeries; increased detection and treatment of diabetic eye disease; and progress in reducing trachoma.

“The services are clearly working, but there’s still more work to do,” Taylor says. “There’s still a lack of services in rural and remote areas. Outreach for ophthalmology services needs to be doubled to meet the population need.”

One of the roadmap’s recommendations was that all patients with diabetes receive an annual eye examination and follow up, and that all patients referred for surgery, receive it. 

The 2016 survey found that the proportion of Indigenous people with diabetes who underwent the recommended eye annual check had increased from 20% in 2008 to 53%.

That means that the number of people going blind because of diabetic retinopathy has reduced, according to Tony Coburn, the Indigenous Eye Health Coordinator at CheckUP, a nongovernmental organization based in Queensland.

However, challenges remain, particularly around increasing people’s awareness of diabetes and its health impact.

“There’s always going to be some people that we can’t reach or who don’t manage their diabetes,” Coburn says.

Experts stress the importance of providing culturally sensitive health services and clear referral pathways to achieve Australia’s vision of closing the eye health gap by 2020.

“There’s still a lack of services in rural and remote areas.”

Hugh Taylor

Clarke knows first-hand how critical it is to provide Aboriginal-specific health resources and how challenging it can be to prevent people from falling through the cracks.

“Let’s say a patient has missed an appointment. If the appointment doesn’t get rescheduled, we need to do that back-up call to try to get them back in to the clinic and to find out what the barrier was,” she says.

“It’s about providing education and support so that patients can make informed decisions about their eye health.”

Clarke says the co-operative had been successful in getting people more engaged with eye health. “If people have someone who is championing eye health, then it’s easier to keep track of the people affected. That’s how systems can work together.”

Lovett agrees, stressing the importance of communicating effectively with patients to “make sure they’re ready, prepared and understand” what their eye examinations will entail.

The most shocking finding from the 2008 eye health survey was the persistence of trachoma in some Indigenous communities. The bacterial infection causes blindness if untreated.

“This has been a national disgrace for 40 years,” Taylor says. “We’re going to eliminate trachoma.”

Since the implementation of the roadmap, trachoma infection rates among children aged 5–9 years across affected communities have fallen from 21% in 2008 to 3.8% in 2017.

Trachoma has been eliminated in 150 communities, but still persists in about 60. The key reason for the improvement has been the full implementation of WHO’s SAFE strategy: surgery for trichiasis; antibiotic treatment; facial cleanliness; and environmental improvements. The key health promotion message has been “Clean Faces, Strong Eyes” and the use of a goanna mascot, Milpa, for campaign events. 

Professor Marcia Langton, foundation chair of Australian Indigenous Studies at the University of Melbourne, believes that the promotion of prevention strategies for children has been critical, specifically getting kids to wash their faces every day.

“Imagine you’re a five-year-old in school and you can’t see the blackboard, because your eyeballs are so scratched up and you’re going blind. That condition should not exist,” Langton says. 

“It’s a health crisis that needs a complex intervention to ensure that this easily eradicable disease disappears from the Aboriginal population.”

Taylor agrees that many elements need to be in place to address the problem, including improving the environment in which many Indigenous people live.

“We need to make sure that kids have clean faces, and clean and functional washing facilities, like clean bathrooms. However, families live in rental houses and there can be huge delays in getting blocked drains and toilets fixed.

“We’re working hard with various government departments to break down the silos and get these facilities working.”

Looking ahead, as Australia strives to close the gap for vision by 2020, Taylor is more committed than ever to get all the recommendations implemented and to eradicate trachoma once and for all.

“The goal is to have all this work done by 2020. Is it achievable? Absolutely!” 

**Figure:**
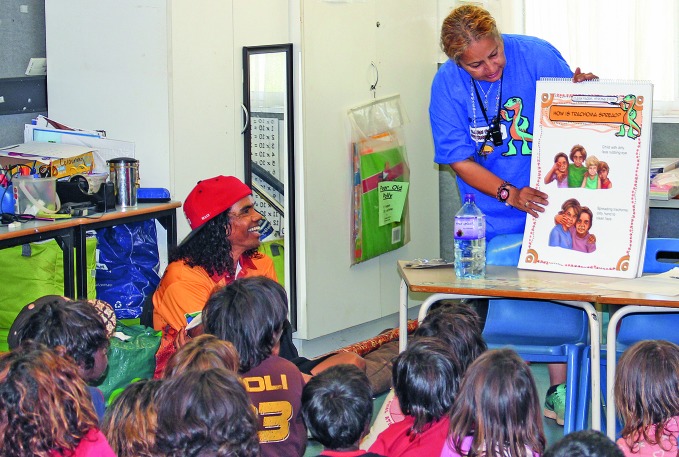
Staff member from the University of Melbourne talks to children about the importance of clean faces, with the support of a community member.

Indigenous Eye Health University of Melbourne

